# The impact of DNA methylation as a factor of Adverse Pregnancy and Birth Outcomes (APBOs): a systematic review protocol

**DOI:** 10.1186/s13643-023-02416-w

**Published:** 2024-01-02

**Authors:** Innocent Moagi, Lawrence Mabasa, Sonto Maria Maputle, Duduzile Ndwandwe, Ndidzulafhi Selina Raliphaswa, Lizzy Mutshinyalo Netshikweta, Thivhulawi Malwela, Amidou Samie

**Affiliations:** 1 Faculty of Sciences, Engineering and Agriculture, Department of Biochemistry and Microbiology, University of Venda, Private Bag X5050, Thohoyandou, 0950 South Africa; 2 Biomedical Research and Innovation Platform (BRIP), South Africa Medical Research Council, Tygerberg, P.O Box 19070, Cape Town, 7505 South Africa; 3 Faculty of Health Sciences, Department of Advanced Nursing Sciences, University of Venda, Private Bag X5050, Thohoyandou, 0950 South Africa; 4 Cochrane South Africa, South Africa Medical Research Council, Parow Valley, Cape Town, 7501 South Africa

**Keywords:** Epigenetics, DNA methylation, Preterm birth (PTB), Low birth weight (LBW), Sepsis, Adverse pregnancy outcomes, Intrauterine growth restriction (IUGR)

## Abstract

**Background:**

Deoxyribonucleic acid (DNA) methylation is one of the epigenetic modifications that has gained a lot of interest as a factor influencing fetal programming and as a biomarker for adverse pregnancy and birth outcomes (APBOs). Epidemiological studies have demonstrated that DNA methylation can result in adverse pregnancy and birth outcomes (APBOs) including miscarriage, intrauterine growth restriction (IUGR), low birth weight (LBW), sepsis, and preterm birth (PTB), which may later result in diseases in adulthood. However, the mechanism by which DNA methylation influences these APBOs remains unclear. The systematic review will assess the association between global and gene-specific DNA methylation with adverse pregnancy outcomes.

**Method:**

The Preferred Reporting Items for Systematic Review and Meta-Analysis (PRISMA) 2020 checklist will be followed when conducting this systematic review. To develop the search strategy the PI(E)COS (population, intervention/exposure, comparator/control, outcome, and study designs) framework will be followed. Thus far, the research team has retrieved 4721 from Cochrane Library, PubMed, Web of Sciences, and MEDLINE. Out of these, 584 studies have been screened for eligibility, and approximately 124 studies meet the inclusion criteria. Pending the search results identified from the grey literature. For identification of unpublished studies in journals indexed in electronic databases, Google Scholar will be used. I.M and A.S will separately extract data from the articles and screen them, if there are any disagreements between I.M and A.S, then the L.M will resolve them. The methodological quality and bias risk of the included studies will be evaluated using the Critical Appraisal Skill Programme CASP) checklist. ${I}^{2}$ and $\chi 2{}$ alpha = 0.10 statistic will be used for assessing statistical heterogeneity between studies. The Grading of Recommendations, Assessment, Development, and Evaluation (GRADE) approach will be used to assess and grade the overall quality of extracted data.

**Ethics and dissemination:**

Ethical approval is not required. The systematic review will assess available literature on possible associations between DNA methylation with adverse pregnancy and birth outcomes (APBOs) including LBW, IUGR, miscarriage, sepsis, and PTB. The findings could help guide future research assessing DNA methylation and other APBOs.

**Systematic review registration:**

PROSPERO CRCRD42022370647.

## Background

Adverse pregnancy and birth outcomes (APBOs) including miscarriage, Low birth weight (LBW), preterm birth (PTB), sepsis, and intrauterine growth restriction (IUGR) are major public health problems and have been linked with a high risk of mortality and morbidities during both the neonatal period and later in life [1–4]. The majority of these APBOs are associated with maternal exposure to genetic and environmental factors during pregnancy. Pregnancy is a vital period of plasticity wherein maternal exposure to multiple environmental, behavioral, and hereditary factors may significantly affect fetal development as well as the mother’s health [5, 6].

Epidemiological studies have shown that intrauterine exposure to adverse environmental factors is associated with adverse pregnancy and birth outcomes, which may increase the risk of developing chronic diseases later in life [7] These studies were inconsistent with the Developmental Origins of Health and Disease (DOHaD) hypothesis. Intrauterine exposure to these factors may also influence offspring’s health later in adulthood, thus influencing susceptibility to long-term risk of chronic diseases from the neonatal period to adulthood [8–11]. Hence, the idea of fetal programming [12–14]. Researchers have shown that perinatal nutrition has a significant impact on fetal programming and pregnancy and birth outcomes [15, 16].

The accurate diagnosis and prognosis of the adverse pregnancy and birth outcomes (APBOs) including PTB, LBW, sepsis, IUGR, and miscarriage remain a big challenge or difficulty, as the majority of these APBOs may share similar clinical signs and symptoms [17–20]. Therefore, there is a need for understanding the etiology and the underlying molecular mechanism behind these APBOs as well as identifying the biomarkers that could be useful for the diagnosis of APBOs during early pregnancy [21, 22]. Molecular mechanisms such as epigenetic modifications, have gained a lot of interest in the identification of potential diagnostic biomarkers for an increased risk of experiencing these adverse pregnancy and birth outcomes (APBOs). This is due to their specificity, prognostic efficacy, and sensitivity when compared to protein expression-based techniques [23, 24].

Epigenetics is the study of inheritable genetic changes that can affect gene expression, without altering DNA sequence. These alterations include DNA methylation, non-coding Ribonucleic acid (RNA) regulation, histone modification as well as chromatin remodeling [1, 9, 13]. Among epigenetic modification, DNA methylation (DNAm) which involves the addition of a methyl group to the cytosine nucleotide of the cytosine-guanine (CpG) dinucleotides, is the well-researched epigenetic mechanism [10, 25]. Enzymes known as DNA methyltransferase act as catalysts and S-adenosyl-methionine as the methyl donor during this process of DNA methylation [23, 26].

Studies recently have made a huge breakthrough on how DNA methylation influences fetal programming and APBOs [1, 27]. For instance, studies have reported on the association of both global and specific-gene DNA methylation with APBOs including IUGR, PTB, LWB, miscarriage, and sepsis separately [20, 22, 28]. In these studies, APBOs were associated with either hypermethylation or hypomethylation of certain genes [21, 28]. Furthermore, studies demonstrated the association between DNA methylation of nuclear receptor subfamily 3 group C member 1(*NR3C1*)*,* long interspersed nuclear element (*LINE-1*), calcitonin-related polypeptide alpha (*CALCA*), and insulin-like factor 2 (*IGF2*) genes with APBOs [10, 19, 29].

Systematic reviews have explored the associations between gene-specific epigenetic modifications of IGF-related genes, *NR3C1,* and Hydroxysteroid 11-beta dehydrogenase type 1/2 (*HSD11 B1/2*) and several APBOs. Notably, these reviews did not examine the impacts of global DNA methylation on APBOs [30, 31]. Furthermore, a systematic review has comprehensively analyzed the literature on the association between DNA methylation signature with PTB in black American women but has not extended to the global population [32]. However, to our knowledge, there are no existing systematic reviews and meta-analyses that have aimed to evaluate the association between both global and gene-specific DNA methylation with APBOs as well as sepsis, all at once. Therefore, the reason for this review is to search for studies addressing the association between DNA methylation and specific APBOs, including LBW, PTB, miscarriage, sepsis, and IUGR. Thus, exploring the impact of environmental factors on DNA methylation, investigating the underlying molecular mechanism by which DNA methylation modifications contribute to the occurrence of APBOs, and identifying potential DNA methylation biomarkers associated with these specific APBOs. The findings from this review will not only contribute to the ongoing efforts to improve both maternal and neonatal health outcomes by shedding light on genetic factors that may influence APBOs but will also provide the knowledge necessary to guide future research and inform clinical strategies aimed at preventing the impact of APBOs.

### Primary objective

This systematic review’s main objective is to assess the relationship of both global and gene-specific methylation status with adverse pregnancy and birth outcomes including intrauterine growth restriction, miscarriage, sepsis, preterm birth, and low birth weight.

### Specific objective

To investigate the association between both neonatal and maternal DNA Methylation status at birth with adverse pregnancy and birth outcomes.

## Methods

### Protocol and registration

The Preferred Reporting Items Systematic Review and Meta-Analysis Protocol (PRISMA-P 2015) guideline will be followed when conducting this systematic review and meta-analysis, which is very crucial in improving the integrity of this review [33]. For this systematic review protocol, a filled-out PRISMA checklist has been provided in the form of a Word document. The protocol used in this systematic review was adopted from the already published systematic review protocol by Vanterpool et al. (2016) and it was submitted for registration in the international prospective register of systematic review PROSPERO (CRD42022370647) [34].

### Eligibility criteria

For inclusion in the review, studies will be screened based on the criteria outlined below. Inclusion will be determined by adherence to the PI(E)COS framework: types of studies, study population, intervention/exposure(s), comparator, and outcomes.

### Types of studies

Observational studies such as cross-sectional studies, prospective cohorts, case–control, and retrospective cohorts focusing on the association between DNA methylation with APBOs will both be considered for the systematic review and meta-analysis. The systematic review will include studies that used either placental samples, the mother’s peripheral blood, neonatal cord blood, or urine samples. Studies published with any language that Google can translate to English will be considered as well as systematic reviews and meta-analyses meeting the inclusion criteria.

### Population of interest

For inclusion in the review, only studies that examined the association between DNA methylation with either LBW, PTB, sepsis, miscarriage, and IUGR in women who are pregnant and their newborns regardless of gender and ethnicity will be considered.

### Intervention/exposure (s)

The exposure of interest will be the DNA methylation status of the participants. There are no interventions that will be reviewed.

### Comparator

The comparison group will include neonates without any complications after birth and women not known for possible confounders (smoking, age, and alcohol).

### Outcomes

The main purpose of this systematic review is to determine whether DNA methylation is associated with adverse pregnancy outcomes. Adverse pregnancy outcomes are any complications that occur during pregnancy, labor, delivery, or 6 weeks after delivery (postpartum period) [35]. For the interest of the systematic review, the following primary and secondary pregnancy and birth outcomes will be taken into consideration based on their occurrence:

### Primary outcomes

*DNA methylation* level of the specific gene in pregnant women and neonates.

*Preterm birth* is birth before 37 complete weeks of gestation (this includes very preterm, moderate preterm, and extremely preterm) [36].

*Miscarriage*, which is a spontaneous loss of pregnancy before 20 weeks [33].

### Secondary outcomes

Low birth weight less than 2500 g (LBW) [36].

When the fetus in the womb is not developing or growing as expected or when the anticipated fetal weight is less than the 10th percentile at birth. This condition is known as intrauterine growth restriction (IUGR) [33].

*Neonatal sepsis* is a systemic condition usually caused by bloodstream bacterial pathogens which is characterized by pro-inflammatory and anti-inflammatory responses, occurring in neonates (particularly PTB and LBW). It is divided into two categories based on the timing of the infection. Thus, early onset sepsis (EOS) which occurs within 72 h of life, and late-onset sepsis (LOS) which occurs after 72 h of life [17–19].

### Settings

There will not be any time and geographical restraints.

### Exclusion criteria

Studies focusing on animals as well as narrative reviews will be excluded from the systematic review. Studies that did not adhere to any PI(E)COS framework will not be considered in the systematic review. Studies examining the relationship between DNA methylation and APBOs in animals.

### Information source and search strategy

For inclusion in this review, the literature search for systematic review will be conducted on the following electronic databases: MEDLINE, Cochrane Library, and PubMed. To ensure comprehensive coverage, reference lists as well as screening citations of the included studies will be manually searched using search engines such as Google Scholar and Web of Sciences. Searching for grey literature, Google Scholar will also be used to identify published articles. The first search approach in PubMed will involve the mixture of Medical Subject Headings (MeSH) and free text search words relating to birth and pregnancy, DNA methylation, epigenetics, intrauterine growth restriction/retardation, low birth weight, miscarriage, and preterm birth. For search in other electronic databases including Cochrane Library and MEDLINE, we will adapt the search strategy used in PubMed with some adjustments. Thus, to remove any contradictions that can affect data extraction. The search terms will be then combined using Boolean operators. The second search technique will focus on the grey literature thus identifying more studies that are not published in journals indexed in Cochrane Library, MEDLINE, and PubMed. The following is the search strategy conducted on PubMed that will be adopted by the reviewer for searching in other databases:

("infant, newborn"[MeSH Terms] OR "fetus"[MeSH Terms] OR "pregnancy"[MeSH Terms] OR "fetal"[Text Word]) AND ("epigenomics"[MeSH Terms] OR "epigenomics"[MeSH Terms] OR "dna methylation"[MeSH Terms]) AND ("pregnancy complications"[MeSH Terms] OR "adverse pregnancy outcomes"[Text Word] OR "premature birth"[MeSH Terms] OR "infant, low birth weight"[MeSH Terms] OR "premature"[Text Word] OR "fetal growth retardation"[MeSH Terms] OR "abortion, spontaneous"[MeSH Terms] OR "sepsis"[MeSH Terms] OR "neonatal sepsis"[Text Word]).

### Study selection

**Fig. 1:**
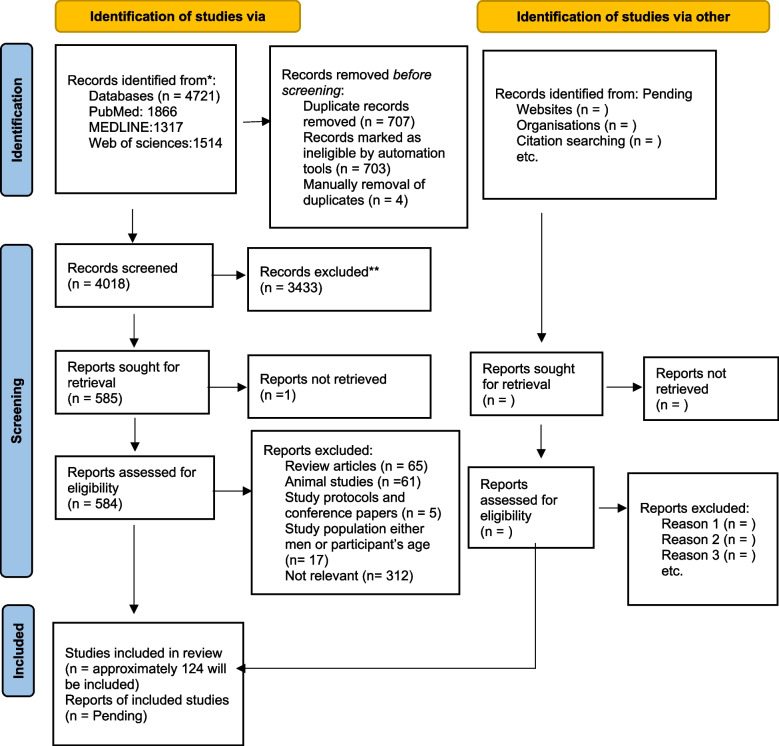
The PRISMA (Preferred Reporting Items for Systematic Review and Meta-Analyses) flowchart for study selection, including preliminary results

All studies identified from electronic databases (PubMed, Web of Sciences, MEDLINE, and Cochrane Library) were combined and imported onto a Mendeley Desktop file. Thus far the researchers have retrieved 4721 from the above-mentioned electronic databases, 584 studies have been screened for eligibility, and approximately 124 studies may be included in the review. Pending the search results from the grey literature. Hence, grey literature studies will be manually entered into the Mendeley Desktop file. The duplicate publications will be first detected and removed automatically using the Mendeley reference manager. For the screening, two reviewers will screen titles and abstracts and the full text of potentially relevant articles. If there are any disagreements between I.M and A.S whether the study is to be included, a discussion will be made with the L.M to resolve the differences. Figure 1 shows the PRISMA flowchart that will be used in summarizing the whole process of study selection, including preliminary results.

### Data collection process

To ensure that the appropriate data for the systematic review is gathered, a structured form with the following descriptive details: author’s information (name and publication year), country of author, type of study, types of samples, characteristics of the participant, investigated genes, DNA methylation techniques, pregnancy outcomes as well as the statistical method used to analyze data will be created. Then if there are any disagreements and conflicts between I.M and A.S, they will be resolved by discussing with the L.M. In case some information is missing from the individual study, there will be efforts to contact the primary author (with a maximum of three email attempts) to obtain the missing data.

### Risk of bias in individual studies

The first and second reviewers will evaluate the study’s methodological quality and bias risk of the studies using the Critical Appraisal Skill Programme (CASP) tool [37]. CASP learning and development opportunities tool that is a part of the Oxford Centre for Triple Value Health Ltd (3v) portfolio that aims to support the development of critical appraisal skills in the United Kingdom [37]. It is recommended for new qualitative researchers to use CAPS which provides an appraisal checklist for analyzing systematic reviews, cohort studies, clinical prediction rules, economic evaluation, case–control studies, observational studies, randomized control trials as well as diagnostic studies and it is recommended for new qualitative researchers [38]. The World Health Organization (WHO) and Cochrane also support CAPS for qualitative evidence synthesis. In case reviewers one and two disagree, the third reviewer will resolve the discrepancies.

### Data synthesis and analysis

For this review data synthesis and analysis will be conducted separately: (1) narrative synthesis wherein the studies meeting the inclusion criteria will be summarized and discussed and (2) statistical analysis wherein the relationship between DNA methylation and APBOs will be investigated.

### Narrative synthesis

Regardless of whether the meta-analysis is appropriate or not, studies meeting the inclusion criteria will be narratively synthesized. A table summarizing the PI(E)CO characteristics and results of the included studies, thus author name, year of study, study design, participants characteristics, definition of exposure, and outcomes will be developed [39]. Lastly, the bias risk will be assessed for each of the included studies.

### Statistical analysis

For the purpose of the systematic review, it is predicted that there will be variation amongst the included studies based on methodological variability (diversity in risk of bias and study design) and clinical variability (diversity in PI(E)CO) [34]. Therefore, inverse variance weighting will be used in a meta-analysis to calculate pooled effect estimates [26]. For the assessment of the degree of inconsistency, forest plot for a pooled estimate of the outcomes will be used first. In prospective cohorts, retrospective cohorts, and cross-sectional studies, the risk ratio (RR with 95% CI) will be used to measure the relationship between gene-specific DNA methylation with APOs while the odd ratio (OR with 95% CI) will be used in case–control (Ahn and Kang., 2018). Chi-square ($\chi 2{}$) alpha = 0.10 and ${I}^{2}$ statistic will be used for assessing statistical heterogeneity between studies. Adding to *χ2* and *I*^2^ statistics,* T*^2^ statistics will be reported thus, to determine how widely distributed the true effects are, especially in the case of meta-analyses with a small number of studies.

${I}^{2}$ of 50% as a moderate or substantial heterogeneity as a guide will be considered in the systematic review [26]. For a meta-analysis with absent or low heterogeneity (*I*^2^ < 50%), a fixed-effected model will be used whereas for moderate or severe heterogeneity, random-effects will be performed [23]. The cause of heterogeneity will be investigated using the meta-regression and subgroup analysis. Variables such as study design, study population, sample size, and outcomes will be used to identify the source of heterogeneity [36]. Meta-analyses will be performed using Comprehensive Meta-analysis (CMA) software Version 3.

### Meta-biases assessment

A funnel plot will be used to assess the probability of publication bias in case there are more than 10 publications looking at the association between DNA methylation status with pregnancy and birth outcomes. For the purpose of evaluating potential publication bias in meta-analysis, Egger’s test for funnel plot will be used [40]. In case of less than 10 studies, a cumulative meta-analysis will be performed, with the studies arranged from the largest to the smallest.

### Confidence in cumulative evidence

The overall quality of extracted data will be assessed and graded using the Grading of Recommendations, Assessment, Development, and Evaluation (GRADE) method considering the following factors: limitation in study design, unexplained heterogeneity, inaccuracy of effect estimates, and risk of publication of bias [26, 34, 36].

### Expected outcomes

Several studies have been published on the association between DNAm with pregnancy and birth outcomes. To our knowledge, there are evidence-based and comprehensive reviews published on the association between both gene-specific and global DNAm during pregnancy with pregnancy and birth complications such as PTB, LBW, IUGR, sepsis, and miscarriage all at once. These call out for the need of comprehensive and systematic information about this association, and to identify the knowledge gaps and to guide future research that will explain how epigenetics affect pregnancy outcomes. Therefore, the main reason for the systematic review and meta-analysis will be to compile data or information from the published studies on the association between DNA methylation with APBOs.

### Dissemination

The Preferred Reporting Items Systematic Review and Meta-Analysis Protocol (PRISMA) guideline will be followed in reporting the systematic review protocol. Both the systematic review and the protocol will be part of Moagi’s MSc research dissertation in which A Samie is the main supervisor, L Mabasa and M.S Maputle are the co-supervisors. Before being submitted for publication in a peer-reviewed journal, the findings from the systematic review will be presented at conferences.
